# Simultaneous CMOS-Based Imaging of Calcium Signaling of the Central Amygdala and the Dorsal Raphe Nucleus During Nociception in Freely Moving Mice

**DOI:** 10.3389/fnins.2021.667708

**Published:** 2021-05-21

**Authors:** Romeo Rebusi, Joshua Philippe Olorocisimo, Jeric Briones, Yasumi Ohta, Makito Haruta, Hironari Takehara, Hiroyuki Tashiro, Kiyotaka Sasagawa, Jun Ohta

**Affiliations:** ^1^Division of Material Science, Nara Institute of Science and Technology, Ikoma, Japan; ^2^Division of Information Science, Nara Institute of Science and Technology, Ikoma, Japan; ^3^Advanced Telecommunications Research Institute International, Kyoto, Japan; ^4^Department of Health Sciences, Faculty of Medical Sciences, Kyusyu University, Fukuoka, Japan

**Keywords:** fluorescence imaging, pain perception, CMOS-based imaging, formalin test, nociception

## Abstract

Fluorescence imaging devices have been indispensable in elucidating the workings of the brain in living animals, including unrestrained, active ones. Various devices are available, each with their own strengths and weaknesses in terms of many factors. We have developed CMOS-based needle-type imaging devices that are small and lightweight enough to be doubly implanted in freely moving mice. The design also allowed angled implantations to avoid critical areas. We demonstrated the utility of the devices by using them on GCaMP6 mice in a formalin test experiment. Simultaneous implantations to the capsular-lateral central amygdala (CeLC) and dorsal raphe nucleus (DRN) were proven to be safe and did not hinder the execution of the study. Analysis of the collected calcium signaling data, supported by behavior data, showed increased activity in both regions as a result of pain stimulation. Thus, we have successfully demonstrated the various advantages of the device in its application in the pain experiment.

## Introduction

Fluorescence imaging brain implants have been indispensable to the field of neurobiology. Their use has given much insight of the inner workings of the brain *in vivo* (Grienberger and Konnerth, 2012). There are a number of types of such devices than can be safely used in living, active animals without compromising the process of a study. These animals are genetically encoded to express fluorescent calcium indicators that signal neuron firing, in a process referred to as calcium signaling. Though all types have their advantages, they also have limitations that must be acknowledged when considering their use. Selecting the appropriate tool can mean the success or failure of doing a study.

Microendoscopes have been used in fluorescence imaging of deep brain tissue. They come in multiple configurations, of differing materials and collection methods (Helmchen et al., 2001; Silva, 2017; Ozbay et al., 2018; Scott et al., 2018; Klioutchnikov et al., 2020). Resolution of collected images is enhanced by the inclusion of a focusing element or lens. The lens itself can be the endoscope, such as the gradient refraction index (GRIN lens). The available endoscopes can be rigid, such as the GRIN lens or silica-containing fibers, but have been miniaturized enough to be of practical use (Levene et al., 2004; Flusberg et al., 2005). These properties provide minimal invasiveness, but also limit mobility since use of these tools require restrained animals. Movement is necessarily restrained because of the fixed sizeable tabletop equipment required for the excitation light source and fluorescence emission collection.

Miniature head-mountable integrated microscopes are an innovation on the use of microendoscopes (Ziv and Ghosh, 2015; Resendez et al., 2016; Liberti et al., 2017; Jacob et al., 2018). They can be used to acquire high resolution images of brain activity from freely moving animals. The implanted component is a cylindrical lens (e.g., GRIN lens) that refracts light to the microscope module secured on an animal’s cranium. Though miniaturized, the sheer bulk needed to accommodate the components gives the device some weight, more than 2 grams in some cases (Helmchen et al., 2001; Ozbay et al., 2018; Scott et al., 2018; Klioutchnikov et al., 2020). This entails consideration for the weight burden on smaller experimental animals, like mice, and the possible effects on brain activity (Ziv and Ghosh, 2015).

**FIGURE 1:**
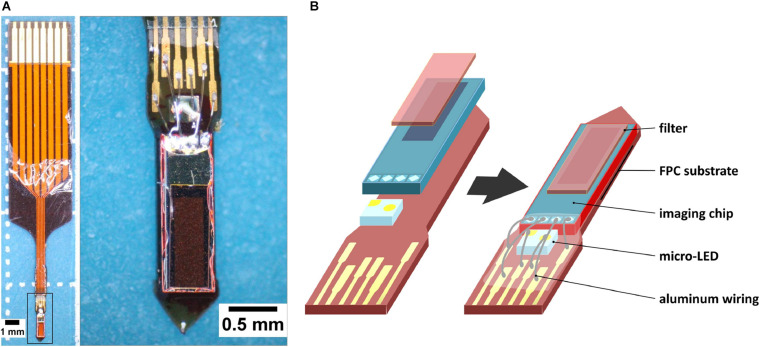
CMOS-based implantable needle-type device for fluorescence imaging. **(A)** Left: Image of the needle-type implantable device as seen under a microscope (28× magnification). This device has undergone all steps of the fabrication process; Right: Higher magnification of the imaging device (from selection, 45×). **(B)** Partial breakdown of the device (left), and the placement of its components (right).

Our lab has developed a needle-type implantable device that has the advantages of the aforementioned devices (Figure 1A; Ohta et al., 2017; Rustami et al., 2020; Sunaga et al., 2020). Our device uses a complementary metal-oxide semiconductor (CMOS)-based image sensor chip (imaging area of 120 by 40 pixels, 7.5 μm per pixel, hence 300 × 9,000 μm) and a blue-light micro-LED (for green fluorescent protein (GFP) excitation), mounted on a thickened flexible printed-circuit substrate (FPC) (Figure 1B). It has a width of 0.7 mm, a thickness of 0.2 mm, an insertion allowance of up to 4.5 mm, and an average weight of 26.6 mg. Its small features and rigidity prevent excessive tissue damage, allow simultaneous implantation of another device, and make possible angled implantations to avoid critical areas and reach difficult sites. Though simultaneous use is still possible with the use of miniature microscopes (de Groot et al., 2020), our devices are much lighter, providing less of a burden on the test animal. The sensor chip allows for imaging of calcium signaling fluorescence, but not to the same resolution as microscopy imaging.

In this study, two of these implantable CMOS-based imaging devices were simultaneously used in mice induced to experience pain via subcutaneous formalin injection. Pain is an immediate and powerful aversive event that has prominent related behavior and can usually activate relevant brain sites with very low latency (review by Woolf, 2010). The devices are implanted adjacent to the capsular-lateral subsection of the central amygdala (CeLC) and the dorsal raphe nucleus (DRN). The central amygdala has been regarded as a center to the processing of pain signals (Bernard et al., 1989; Neugebauer et al., 2004; Ossipov et al., 2010; Veinante et al., 2013) and responsible for pain-related behavior as an output center of the amygdala (Ji et al., 2017). Meanwhile, the DRN has serotonergic connections with the CeLC (Peyron et al., 1997) and has been implicated in the process of pain modulation (Wang and Nakai, 1994; Stamford, 1995; Li et al., 2017; Lopez-Alvarez et al., 2018). Together, they are part of a system responsible for processing aversive stimuli and stress (Spannuth et al., 2011; Groessl et al., 2018; Ren et al., 2018; Zhou et al., 2019).

This study’s aim is twofold. First, it is to demonstrate that the simultaneous use of two devices on an unrestrained animal is without risk and also allows the animal behave unhindered. Second, is to provide useful inner-brain imaging data of the pain processing circuitry. This is accomplished through calcium imaging at the relevant sites. Overall, we aim to show that our device can complement established brain imaging implants while also providing unique advantages.

## Methodology

### Device Fabrication

The CMOS imaging chips, designed by our laboratory, were cleaned by submersion in acetone (Fujifilm Wako), twice, and then in isopropanol (Fujifilm Wako) for 5 min each. After drying, they were each mounted on a polyimide, gold-circuit-printed FPC substrate (Taiyo Industrial) taped on a glass slide using a thin layer of epoxy resin [low viscosity epoxy resin Z-1 (N), CraftResin]. The blue-light-emitting micro LEDs (ES-VEBCM12A, Epistar), with a central emission wavelength of 470 nm, were also mounted in the same fashion.

The blue-light filter was prepared by first dissolving Valifast Yellow 3150 dye (Orient Chemical Industries) with cyclopentanol (1:1, w/w) overnight in a light-proof vial. In the same container, the mixture was mixed with Norland Optical Adhesive 43 (Norland Products) (2:1, w/w) using a vortex. The resulting adhesive mixture was spin coated (Spincoater model: 1H-D7, Mikasa) on a silicon-coated [CAT-RG catalyst and KE-106 silicone (Shin-Etsu Chemical), 1:10, w/w] cover slip with a size of 23 mm × 23 mm under the following setting: 3 s to 500 rpm–5 sec at 500 rpm–5 s to 2,000 rpm–20 sec at 2,000 rpm–5 s to 0 rpm. The spin coated material was immediately heated on a hot plate at 100°C for 30 min then left to set in room temperature overnight. The filter film was cut with an Nd: YAG laser (Callisto VL-C30RS-GV, TNS Systems) to make a grid of 10,000 × 3,500 μm sheets.

Cut filter sheet were manually placed over the entire imaging area of the CMOS chips. The device was baked in a vacuum oven (AVO-250NS, ETTAS) for 2 h at 120°C and left to cool.

Using a needle, red resist resin (ST-3000L, Fujifilm) was applied on the sides of the CMOS chip. This resin prevented entry of stray blue LED light through the sides of the chip.

The CMOS chip and the LED were connected to the circuitry of the FPC with micro aluminum wires (Tanaka Electronics) using a wire bonder (7400C-79, West Bond). The wiring was sealed by a protective cover of epoxy resin [low viscosity epoxy resin Z-1 (N), CraftResin] and left to set overnight.

The processed device was incised off from the excess FPC material with a blade. The sides at implantable end of the cut FPC substrate of the device were coated with a thin layer epoxy [low viscosity epoxy resin Z-1 (N), CraftResin] to cover jagged edges.

The devices were lined and bound together with a ribbon of Kapton tape and were enclosed in a parylene coater (PDS 2010, Specialty Coating Systems). They were coated with 5 grams of dichloro-c-cyclophane (GalentisS.r.l.). The thickness of the parylene deposition layer is about 2.5 μm. This transparent, long-lasting coating serves as a bio-protective sheath to prevent infiltration of biological substances into the device that can damage the circuitry.

### Implantation

All animal handling procedures were approved by the Nara Institute of Science and Technology (NAIST) Animal Committees, and were performed in accordance with the institutional guidelines of the animal facilities of NAIST.

**FIGURE 2:**
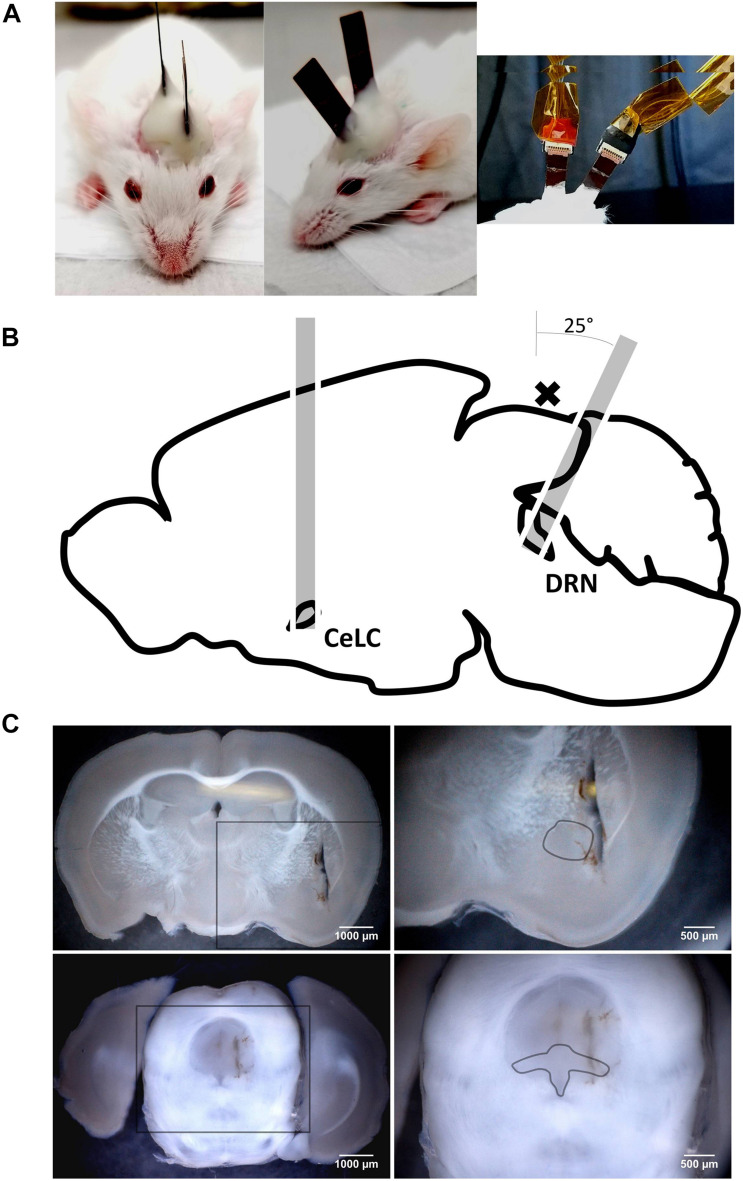
The implanted mouse and the paths of the implants to their targets. **(A)** Left and Middle: A mouse that has just underwent double-implantation, under anesthesia. Note the differing angles of the implanted devices; Right: Implanted devices attached to cables used for supplying power and collecting calcium-signaling fluorescence data. **(B)** Paths of implantation through the brain. The cross (x) indicates the location of a large superficial transverse blood vessel on the brain’s surface. **(C)** Coronal sections depicting lesions from implantations and the loci of the brain site targets. Lesions do not reach the dorsal brain surface because the sectioning and the implantation planes are not parallel, more so for the DRN targeting. Upper Left: AP: –1.58 mm, 25×; Upper Right: Zoomed in section from rectangle in upper left image, dashed line approximates the perimeter of the central amygdala (CeA), 45×; Lower Left: AP: –4.36 mm, 25×; Lower Right: Zoomed in section from rectangle in lower left image, dashed line approximates the perimeter of the DRN (dorsal, ventral, and lateral subsections), 45×.

GCaMP6 mice [strain: FVB-Tg(Thy1-GCaMP6)5Shi., provided RIKEN BRC through the National Bio-Resource Project of the MEXT, Japan (Ohkura et al., 2012)], around 2 months of old of either sex, were implanted with two of our CMOS-based imaging devices (Figure 2A). They were positioned adjacent to the CeLC and the DRN, both in the left hemisphere (Figure 2B).

The mice were anaesthetized. Hair was removed from the top of their heads and the scalp was disinfected with 4% clorhexidine (Hibitane). They were restrained to a stereotaxic platform (SR-6M, Narishige) using earbars. A heating pad was provided underneath the animals to stabilize body temperature.

Skin was excised from the dorsal side of the head, just enough to access the implantation sites. The cranium was exposed by clearing away tissue and washing with PBS (Fujifilm Wako). After aligning the bregma and lambda, coordinates for the brain site targets were marked on the cranium [CeLC: AP: −0.8 mm, ML: −3.35 mm (left), DV: −4.0 mm; DRN: AP: −5.56 mm, ML: −0.35 mm (left), DV: −3.31 mm; all DV coordinates counted from the dura]. All coordinates were determined using the Paxinos Mouse Brain Atlas (Franklin and Paxinos, 2008) and calibrated in previous trials. Two small micro-screws were shallowly secured into the cranium, spaced some distance from the marked sites. Small cranial windows of around 1 mm in diameter were created on the targets using a dental drill (Hypertec II, Morita) and were cleansed of debris and blood with PBS.

The implants were gently wiped with 70% ethanol (Fujifilm Wako) using cotton swabs and were secured to stereotaxic manipulators (SM-15M, Narishige). They were slowly implanted into the windows, with the CMOS chip facing medially toward the target, up to the predetermined depth. For the DRN, the path of implantation was angled 25° posteriorly to avoid damaging the major superficial blood vessel lying between the entorhinal cortex and the cerebellum (Figure 2B).

Once the targets were reached, the cranial windows were sealed with silicone elastomer (Kwik-Cast). The exposed cranium and the surrounding incised skin were sealed with dental cement (Super-Bond kit, Sun Medical). The cement was also used for stabilizing and anchoring the implanted device unto the skull surface and the inserted screws. The manipulators and other restraints were removed. The mouse was allowed to recuperate for at least 12 h in a heated enclosure.

### Formalin Test

**FIGURE 3:**
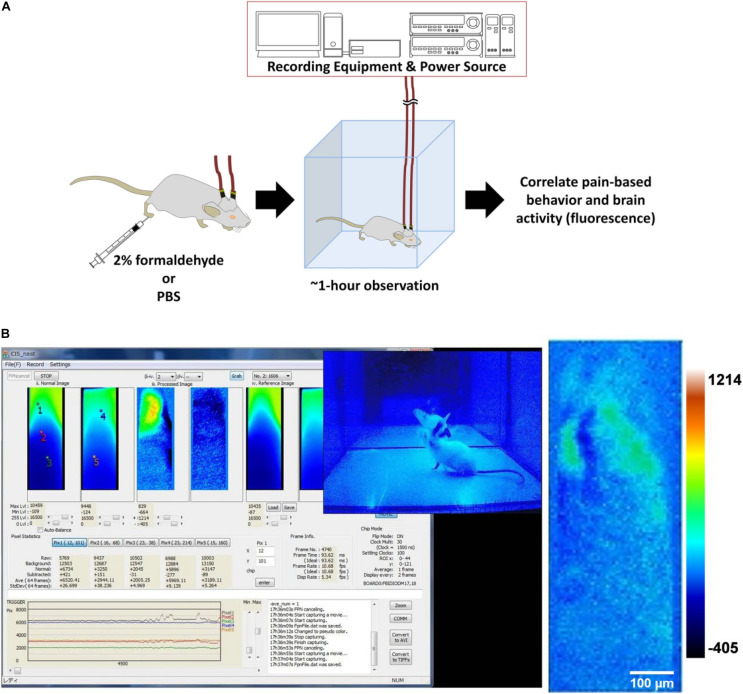
Formalin test with brain activity imaging. **(A)** The experimental design of the modified formalin test. **(B)** Left: Screenshot of the computer monitor during a formalin test. On the screen is the CIS_NAIST software that displays the windows (40 × 120 pixels, 300 × 9,000 μm) for raw, processed, and reference frames of real-time video data, in pairs from left to right. The left of the pair is the fluorescence image from the CeLC, and the right of the pair is from the DRN. Also displayed are the fluorescence level traces at the bottom and frame number information at the middle right. The overlying the CIS_NAIST software window is the video feed of the mouse behavior from the webcam; Right: A sample of a processed image of the CeLC with discernible fluorescent structures. Heatmap values are voltage values that represent ΔF.

A modified formalin test was done with the aim of recording brain activity during pain perception, indicated by fluorescence from calcium signaling due to neuronal action potentials. Behavior attributed to pain perception, such as licking, was used to support the validity of the fluorescence imaging data (Figure 3A).

The whole recording procedure was done within a darkened canvas tent. This is to prevent the interference of light from sources such as illumination lamps for animal handling, interior overhead lighting, and sunlight passing through windows. Light from such sources are of high enough intensity to permeate the very thin skull, and overlying layers, of the experimental mouse. Such filtered light can still be detected by the implanted devices due to the high sensitivity of the CMOS imaging chip. The orbit and the frontal cranial bone are especially exposed to light penetration. Though the parietal cranium bone is covered with reinforcing dental cement, the cement itself is translucent. Implanted mice were anaesthetized with isoflurane (Fujifilm Wako) using an isoflurane pump (410 Anaesthesia Unit, Univentor). The implants were then connected to cables that were then connected to a slip ring. The rotating slip ring prevented entanglement of the cable pair and served as a bridge between the implants and the power sources (6146 DC Voltage Current Source, ADCMT) and collecting equipment (custom-made, NAIST).

After securing the connections, the mice were kept in an observation enclosure and monitored with a webcam (CMS-V37BK, Sanwa Supply). Illumination was under blue-light that was blocked by the blue-light filter and the red resist coating of the device. Their brain activity and behavior were recorded on a personal computer using specialized custom-made software (CIS_NAIST) and a webcam software (Bandicam) (Figure 3B) for a minimum of 10 min to serve as a baseline. Afterward, they were subcutaneously injected using a 30G needle with either 20 μL 2% paraformaldehyde-PBS (Fujifilm Wako) [“Formalin” group (*n* = 3)] or PBS (Fujifilm Wako) [“PBS” group (*n* = 3)] at the plantar side of the right hind-paw, contralateral to the side of the implantations. Brain activity and behavior were recorded for a minimum of an hour. For the duration of the experiment, experimenters vacated the tent to prevent affecting behavior.

At the end of the experiment, the mice were sacrificed with an overdose IP injection of sodium pentobarbital (Somnopentyl, ∼0.2 mL, KS Medical). They were perfused with a tubing pump (TP-10SA, AS ONE) with normal saline (Otsuka) and then 4% formaldehyde (Fujifilm Wako). Their brains were extracted and stored in 4% formalin overnight. Coronal sections of the brains (100 μm) were prepared using a vibratome (Linear Slicer PRO7, Dosaka) and were used to confirm successful targeting of the brain sites (Figure 2C). Data from animals with unsuccessful implantations were disregarded.

### Implanted Brain Temperature Reading

The inner-brain temperature, in Celsius, of an implanted mouse was measured during activation of the blue-light micro-LED at 0.5 mA. Two thermocouples (Cu/constantan (Type T) thermocouple, Muromachi Kikai Co., Ltd., Tokyo, Japan) were bounded unto needle-type devices with Parafilm M (PM-996, Pechiney Plastic Packaging). One thermocouple terminal was located by the LED and another at the device’s insertion tip, serving as a reference site away from the LED. The thermocouples were connected to a microcomputer thermometer (BAT 700 1H, Physitemp Instruments LLC).

Mice were anaesthetized and implantations were done into the DRN of one mouse and the CeLC of another as previously described, but with some differences. After implantation, elastomer sealant was not used as a protective cover. Instead, PBS-moistened pieces of Kimwipe were applied on the exposed region surrounding the implant and were held in place with Parafilm. The animal’s body temperature was allowed to stabilize. Temperature was recorded for 5 min before LED activation, for an hour during activation, and for 5 min afterward.

### Data Processing

Videos of the brain fluorescence activity were collected using CIS_NAIST and screen recordings of the working computer. Images of the fluorescence in the CeLC and the DRN were extracted from the time immediately after the injection of the substance and every 10 min thereafter, until the end of the 1-h observation period. The clearest calcium imaging result was selected as a representative example of each sampling group.

Behavior recorded by the webcam was reviewed and pain-related behaviors were taken note of, specifically the licking of the injected site. Behavior was quantified by tallying the amounts of pain-induced licks on the affected paw per 2.5 min blocks within the 1-h observation period. The results were graphed as a number of licks per time block along the passage of time.

Imaging data was acquired using CIS_NAIST, and saved as RAW files. The data was extracted from the files and analyzed using MATLAB (MathWorks). Custom made codes were written in order to process and visualize the data. For processing, the data was first stored as a 3-dimensional matrix (2D spatial pixel array across time), then separated into two for the CeLC and the DRN data. Afterward, the period of injection was determined from webcam recordings, and also by looking at the offset frames in the averaged data set.

Removal of hum noise was performed by making a column vector containing the average values of each row in the pixel array. The average value of that was computed and subtracted to each element of the column vector. The resulting vector was subtracted to each column in the pixel array. This was done in each frame. That is, given a pixel reading *F*_*t*_(*x*,*y*) at frame *t*, where *x* = 1 : *n*_*x*_, *y* = 1 : *n*_*y*_, the following steps are performed for each *t*:

(1) Form the column vector ${\bar{x}}_{t},$ where ${\bar{x}}_{t}\,\left(y\right)=\frac{1}{n_{y}}\,\sum_{i=1}^{n_{x}}F_{t}\,\left(i,y\right)$ for each *y* = 1 : *n*_*y*_.
(2) Calculate $\bar{F_{t}}=\frac{1}{n_{x}}\,\sum_{j=1}^{n_{y}}{\bar{x}}_{t}\,\left(j\right)$.
(3) Form the column vector *h_t_*, where $h_{t}\,\left(y\right)={\bar{F}}_{t}-{\bar{x}}_{t}\,\left(y\right)$.
(4) The new reading $F_{t}^{*}\,\left(x,y\right)$ is then given by $F_{t}^{*}\,\left(x,y\right)=F_{t}\,\left(x,y\right)-h_{t}\,\left(y\right)$.

The data was normalized (ΔF/F_0_) by getting the average of the frames before injection as the baseline. That is, given *F*_*t*_(*x*,*y*), the baseline *F_0* is given by $F_{0}\,\left(x,y\right)=\frac{1}{t^{*}}\,\sum_{t=1}^{t^{*}}F_{t}\,\left(x,y\right)$, where *t*^∗^ is the frame number before injection. The normalized pixel reading $F_{t}^{*}\,\left(x,y\right)$ is then given by $F_{t}^{*}=\frac{△\,F}{F_{0}}=\frac{F_{t}-F_{0}}{F_{0}}$, where $F_{t}^{*}\,\left(x,y\right)=\frac{F_{t}\,\left(x,y\right)-F_{0}\,\left(x,y\right)}{F_{0}\,\left(x,y\right)}$.

Based on a previously published study (Takehara et al., 2016), the approximate size of neurons was computed and the regions of interest (ROIs) were selected accordingly. Specifically, it was assumed that the maximum distance between a visible ROI and image sensor surface was 100 μm, and therefore; the full-width at half-maximum (FWHM) would increase 3–4 times compared to a distance of 0 μm. Given the soma and device pixel sizes (8–9 μm and 7.5 μm, respectively) and the 4 times increase in soma size due to the distance from image sensor, then the ROI was computed to be around 6 × 6 pixels. Regions that seemed like neurons based on fluorescent activity were selected as ROIs for further analysis. The average of each ROI was plotted and compared against background values outside the ROIs. A scale bar representing 5% change from baseline was generated. A color plot showing the intensity of each pixel in a frame was graphed to visualize the ROIs. The behavioral data was aligned with the calcium imaging data to see their relationship.

Afterward, the first-differenced calcium traces in each ROI were cross-correlated or auto-correlated. First-difference was applied to ensure stationarity of the data. That is, given a calcium trace reading *z_t_* at time *t*, the first-differenced calcium trace △*z*_*t*_ at time *t* is △*z*_*t*_ = *z*_*t* + 1_−*z*_*t*_. First-differencing was implemented using **diff()** function of MATLAB. Cross-correlation between CeLC and DRN ROIs was calculated by shifting the DRN data across time. That is, given the CeLC calcium trace reading *x_t_* at time *t* and the DRN calcium trace reading *y_t_* at time *t*, the non-normalized cross-correlation coefficient *R* at lag *m* is given by

$$R_{x,y}\,\left(m\right)=\left\{ \begin{array}{cc} \sum_{t=1}^{T-m}x_{t}\,y_{t-m} & \text{if}\,m\geq 0\\ R_{y,x}\,\left(-m\right) & \text{if}\,m<0 \end{array}\right..$$

The (normalized) cross-correlation ρ at lag *m* is then given by $\rho_{x,y}\,\left(m\right)=\frac{1}{\sqrt{R_{x,x}\,\left(0\right)\,R_{y,y}\,\left(0\right)}}\,R_{x,y}\,\left(m\right)$. Here, *R*_*x*,*x*_(*m*) and *R*_*y*,*y*_(*m*) represents the non-normalized auto-correlation at lag *m* for *x_t_* and *y_t_*, respectively. The DRN time lag with the highest correlation with CeLC *m*^∗^ = argmax_*m*_ ρ_*x*,*y*_(*m*) was recorded as the best time lag. Cross-correlation analysis was implemented using **xcorr()** function of MATLAB. The three ROIs from CeLC and three ROIs from DRN were cross-correlated as follows: CeLC vs. DRN, CeLC vs. CeLC, and DRN vs. DRN. Therefore, a total of 27 cross-correlations were analyzed per mouse.

To measure the relationship between brain imaging and behavior, the mutual information coefficient (MI) between these variables was computed. Basically, the mutual information coefficient (Cover and Thomas, 2006) between two (discrete) random variables *X* and *Y* is given by

$$I\,\left(X;Y\right)=\sum_{x\in 𝒳}\sum_{y\in 𝒴}p_{X,Y}\,\left(x,y\right)\,\log\left(\frac{p_{X,Y}\,\left(x,y\right)}{p_{X}\,\left(x\right)\,p_{Y}\,\left(y\right)}\right).$$

Similarly, if *X*,*Y* are continuous, then the summation is replaced with integration. However, since brain imaging is a continuous random variable, while the behavior is discrete, MI was measured using an adapted method (Ross, 2014). MI was computed using **discrete_continuous_info_fast()** function of Ross (2014). Since the imaging data was a continuous real-valued dataset, it was binned and averaged across the same 2.5 min window of the behavioral data. This enabled the measurement of mutual information between continuous imaging data and discrete behavioral data, where a higher value indicates more dependence between the two.

Finally, statistical analysis was done using MATLAB. Non-parametric tests were performed: Kruskal-Wallis test for the cross- and auto-correlation analysis and non-parametric two-way ANOVA (Friedman’s test) for the brain calcium imaging and licking behavior relationship analysis were performed. *P*-values less than 0.05 were considered significant. Boxplots with median and interquartile range were graphed also using MATLAB.

## Results

### Brain Recordings: Fluorescence Imaging and Internal Temperature

No notable complications on the welfare of the experimental mice were encountered during the duration of the study. Implantation surgery was accomplished without issues and all the mice recuperated fully the following day. No signs of distress that may have come from the implantation were observed. Furthermore, there were no indications of encumbrance of the head. Post mortem weighing of the cement-bound dual implants, excluding the parietal portion of the cranium, gave an average weight of 0.474 g (*n* = 5). The only signs of discomfort were the pain-related behavior and inflammation reaction of the Formalin group mice after injection and the slight agitation immediately resulting from animal handling.

**FIGURE 4:**
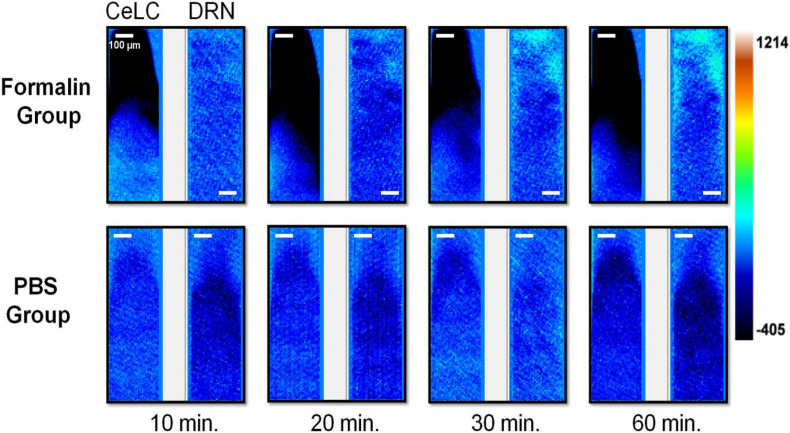
Brain activity fluorescence immediately after injection of formaldehyde or PBS. Neuronal fluorescence from the replicates that demonstrated detectable and distinct signals are presented. Time shown refer to the minutes that has passed after injection of the assigned substance. For all image pairs, CeLC is on the left and the DRN is on the right. Heatmap values are voltage values that represent ΔF.

Images of the calcium signaling from representative examples were chosen (Figure 4). The top right corner of the DRN in the Formalin group had an increasing intensity throughout the time course, from the 20-min mark onward, with distinct and prominent fluorescence. The CeLC of the same group, particularly the lower-left of the imaging area, initially displayed some fluorescence that gradually lessened over time.

On the other hand, the PBS group had more uniform and constant images across time. Slight changes in overall fluorescence can still be seen, such as a brighter DRN and CeLC at the 30-min mark. The changes are quite difficult to spot by eye alone. Therefore, a more quantitative approach was taken.

**FIGURE 5:**
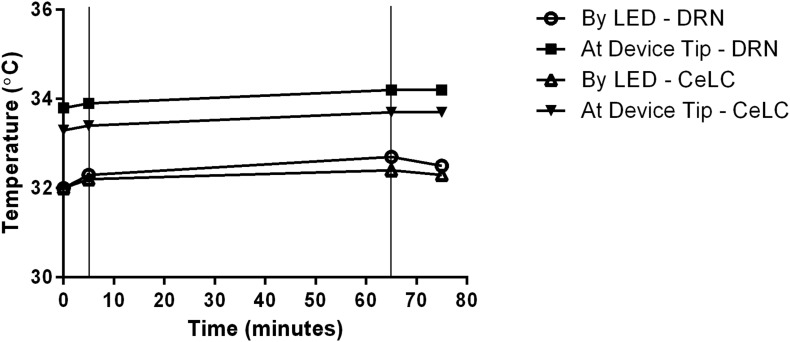
Recorded change of DRN and CeLC temperatures during implantation. Temperature readings were done in the vicinity of the implanted devices, specifically by the LED and at the device insertion tip. Thin vertical lines in the graph indicate start and end points of LED activation time.

Readings in a double-implanted mouse depict very small increase in deep brain tissue temperature during activation of the blue-light micro-LED (Figure 5). At most, there was 0.5°C increase in the portion of the DRN next to the LED. The results also show that proximity to the LED does not necessarily lead to a higher tissue temperature.

### Data Analysis

**FIGURE 6:**
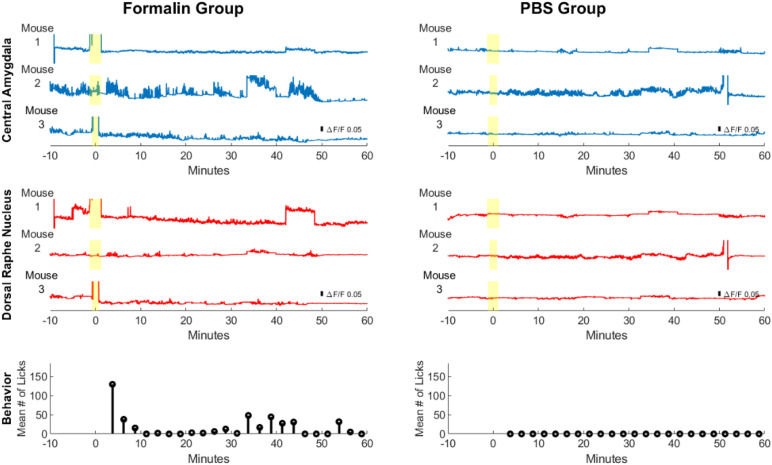
Calcium imaging and behavioral analysis across multiple mice. Brain calcium imaging and behavioral analysis of Formalin- and PBS-injected mice. The yellow highlight marks the injection period. The blue line graphs show calcium measurements of the CeLC across multiple mice, while the red line graphs show calcium measurements of the DRN. At the bottom are the behavioral results for the average number of licks per 2.5 min time block among Formalin mice (*n* = 4) and PBS mice (*n* = 3).

To better visualize changes in CeLC and DRN fluorescence activity, the whole frame average across time was computed in all mice (Figure 6, top and middle). In addition, the paw-licking behavior of the mice was also measured simultaneously (Figure 6, bottom). Due to the small size of the implantable device, both CeLC and DRN can be visualized at the same time in a freely behaving mouse. Based on the ΔF/F_0_ scale bar, the Formalin group generally had higher amplitudes than the PBS group. For example, Formalin Mouse 2 displayed higher fluorescence values compared to PBS Mouse 2 in the CeLC. Furthermore, Formalin Mouse 1 had higher fluorescence intensities than PBS Mouse 1 in the DRN. In addition, the Formalin group had higher frequency of fluorescent peaks for both brain sites. This was most apparent for Mouse 2 and 3 for CeLC and Mouse 1 for DRN. Conversely, the PBS group had graphs that are comparatively flatter. Since this was a proof-of-concept to show that the dual-implantable device works, more mouse samples and further analysis can be done to quantify the difference in amplitudes and frequencies between both groups.

Pain-related behavior was observed from all the mice of the Formalin group, as reflected by the average number of licks. A high number of licks was seen in the initial minutes after injection. The licking behavior then subsided after 10 min. Then, after 30 min licking behavior started to increase again. This bi-phasic response may correspond to the acute and inflammatory phase of formalin injection, as will be discussed later. Interestingly, peaks in licking behavior also corresponded to higher fluorescence activity in the CeLC and DRN. On the other hand, the PBS mice did not display any hindpaw-licking behavior. These results confirm the successful execution of the formalin test.

**FIGURE 7:**
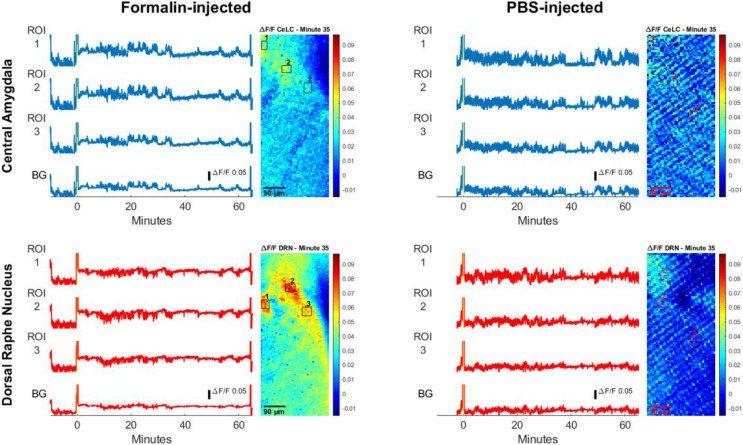
Calcium traces of specific regions of interest (ROIs). Calcium measurement of Formalin-injected mouse and PBS-injected mouse. The yellow bar on the line graph marks the injection period. The blue line graphs show measurements in the CeLC, while the red line graphs show measurements of the DRN. The background values (BG) are the average of all the pixels outside the ROIs. Beside the line graphs are images of sample frames where ROIs are enclosed in black or red boxes.

Another two mice were selected as representatives, one per sampling group, to visualize analysis of multiple regions of interests (ROIs) (Figure 7). Slight differences in timing and intensity of fluorescence can be seen in ROIs of the same brain region. For instance, in the Formalin-injected mouse, DRN ROI2 displayed more fluorescence activity than DRN ROI1 at the 20-min mark. It is also observable that the ROIs of the DRN have higher and more distinct spikes than the background. Furthermore, ROI 1 and 2 of the CeLC had higher fluorescence amplitudes than ROI 3. However, CeLC ROI 3 still had higher fluorescence than the CeLC background values.

On the other hand, the PBS-injected mouse had ROI values that are relatively similar with the background. Similar to Figure 6, the PBS mouse had a more constant fluorescent activity where its peaks do not deviate too far from the average noise value. The peaks of the Formalin-injected ROIs were more distinct and had higher amplitudes compared to its baseline level before injection.

**FIGURE 8:**
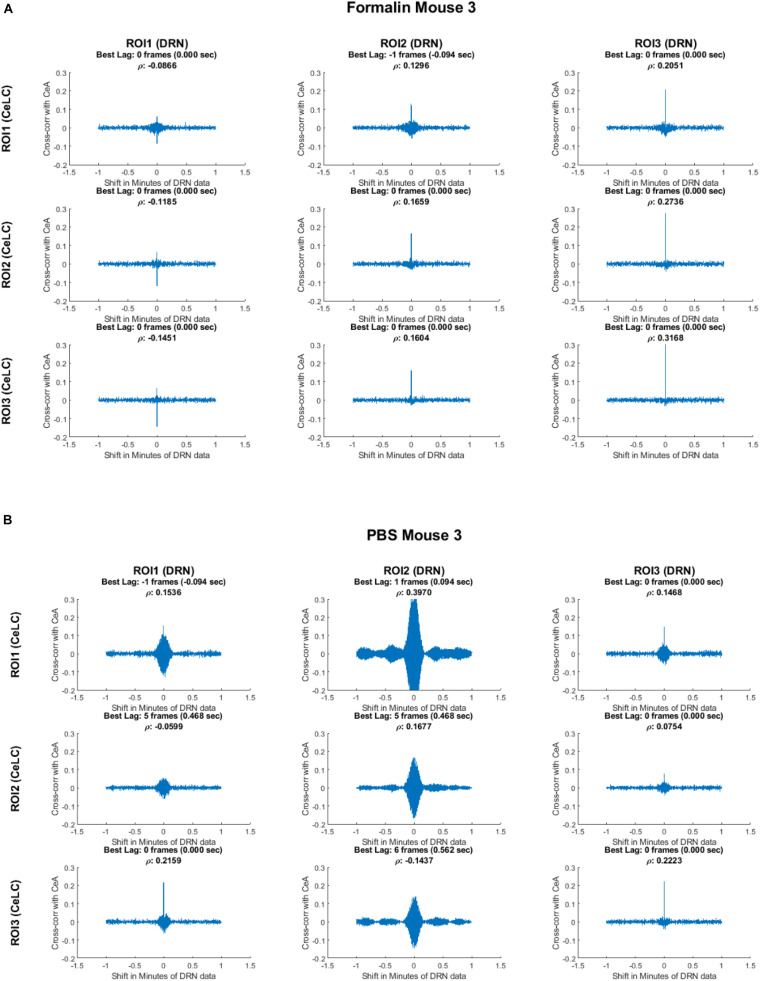
Cross-correlation analysis of two representative mice. Top: Formalin-injected mouse; bottom: PBS-injected mouse. The figure shows the cross-correlation of the first-difference of CeLC fluorescence imaging data with DRN data. The correlation between CeLC and time-shifted DRN (across varying lags) is measured. Peaks indicate high cross-correlation at that specific shift or time lag of the DRN data. The time lag with the highest cross-correlation coefficient (ρ) is indicated at the top of each graph.

The relationship between each ROI was explored. The small size and large field of view of the device made it possible to measure cross-correlation of multiple ROIs within and between the CeLC and DRN. First, cross-correlation analysis of the Formalin-injected mouse showed distinct and clear peaks mostly at time lag 0 (Figure 8). The only exception was the cross-correlation between CeLC ROI 1 and the DRN ROI 1. The data indicated that when the first-differenced DRN data is shifted 1 frame back, then the correlation with first-differenced CeLC data increases. This means that when the CeLC fluorescence increases, there is a positive correlation that the DRN fluorescence will also increase after 0.094 s. However, this was only true for one ROI of the CeLC. The other two ROIs of the CeLC showed a definite peak at 0 time lag, which indicated that the cross-correlation was highest at 0 s delay.

In contrast, the PBS-injected mouse had less clear and less distinct cross-correlation peaks. It can be seen that most of the cross-correlation graphs are broader, especially for DRN ROI 2. Furthermore, the best time lag is less consistent among the ROIs, with one pair having the highest cross-correlation at 6 frames (0.562 s) and two pairs of ROIs at 5 frames (0.468 s). This may indicate less synchronization between CeLC and DRN neuronal firing in PBS-injected mouse.

**FIGURE 9:**
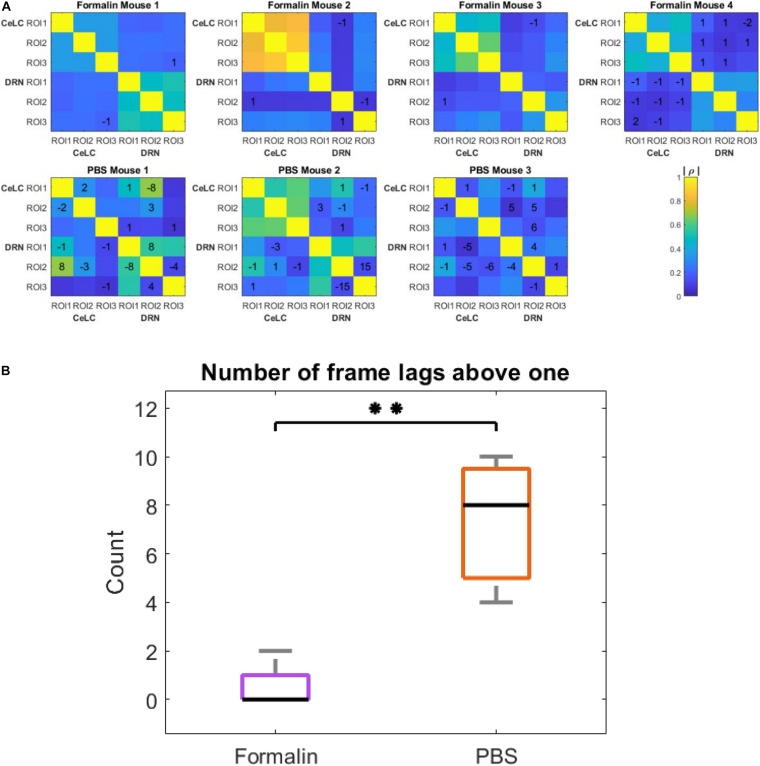
Cross- and auto-correlation analysis in all mice. **(A)** Heatmap showing time lags with the highest cross-correlation coefficients. ROIs from CeLC and ROIs from DRN in each mouse were cross-correlated. The values written indicate the time lag of first-differenced DRN data in frame number that showed the highest cross-correlation with the original unshifted first-differenced CeLC data. No value means the best lag was at 0. The color indicates the cross-correlation coefficient (ρ)from low (blue) to high (yellow) in absolute values. **(B)** Box plot of the number of frame lags that were above 1 in both treatment groups. Kruskal-Wallis test was performed and a significant difference was computed (^∗∗^*p* = 0.00773, α = 0.05).

To provide more insight and see if the results are consistent, the cross-correlation of ROIs in each mouse was analyzed (Figure 9). Majority of the peak cross-correlations was at time lag 0 (Figure 9A). However, it can be observed that PBS mice had several non-zero and non-one time lags. For example, PBS Mouse 1 had a high cross-correlation (ρ = 0.69) at −8 frames when analyzing between its CeLC ROI 1 and DRN ROI 2. Then its CeLC ROI 2 and DRN ROI 2 had the best lag at +3 frames.

Furthermore, even within the DRN, there was a high cross-correlation at 8, 15, and 4 frame lags for PBS Mouse 1, 2, and 3, respectively. On the other hand, more cross-correlations at time lag 0 can be seen in Formalin-injected mice. For instance, when comparing within the same brain region (i.e., CeLC vs. CeLC or DRN vs. DRN) all ROIs displayed a best time lag of 0, with the exception of DRN ROI 2 and 3 of Formalin Mouse 2. Taken together, this may indicate less synchronization within the DRN of PBS-injected mice.

To compare the difference between the number of frame lags higher than 1 for each mouse group, a two-tailed unpaired *t*-test was performed. The number of frame lags higher than 1 was statistically higher in the PBS group compared to the Formalin group (*p* = 0.00773, Unpaired *t*-test). This confirmed the hypothesis that PBS-injected mice had a higher number of ROIs that were not as synchronized compared to the Formalin-injected mice.

**FIGURE 10:**
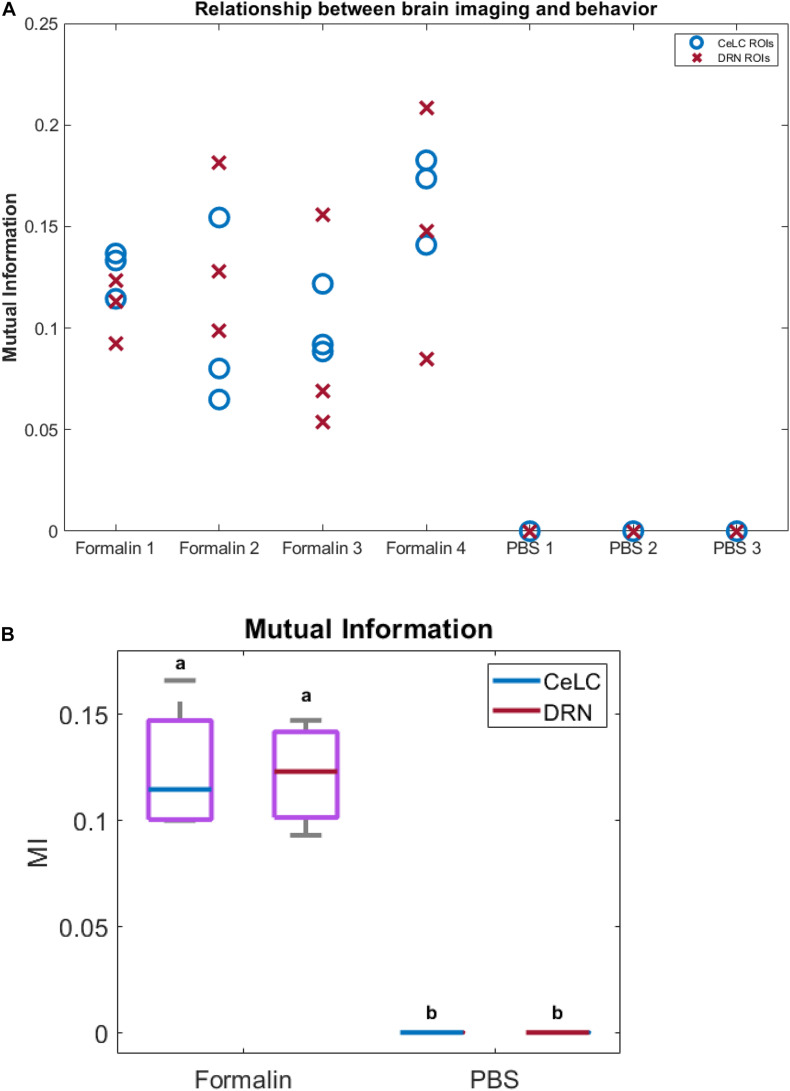
Relationship between brain calcium imaging and licking behavior. Relationship between brain calcium imaging and licking behavior. **(A)** Plot of mutual information between imaging and behavior. Blue circles indicate ROIs from CeLC, while red crosses indicate ROIs from DRN. **(B)** Box plot of the mutual information (MI) when ROIs in each mouse were averaged. PBS mice showed almost 0 MI with behavior, while Formalin mice showed higher MI values. Non-parametric two-way ANOVA showed significant difference between Formalin and PBS groups (*p* < 0.05, α = 0.05), but no significant difference between CeLC and DRN within each group.

Finally, to measure the relationship between the brain calcium imaging data and licking behavior of mice, we calculated their mutual information (MI). The MI between calcium imaging of PBS mice and their licking behavior is close to 0, showing a lack of a relationship. On the other hand, the Formalin mice displayed higher MI levels across different ROIs and brain areas (Figure 10A). This was further confirmed using two-way ANOVA, wherein the Formalin group was significantly different compared to the PBS group (*p* < 0.05). However, no difference was detected between CeLC and DRN within each group.

By using this MI metric, the capability of the dual-implantable device to correlate brain activity and behavior was demonstrated. This showed the potential of the device for use in further experiments and analysis.

## Discussion

We have demonstrated that the use of our device in a double-implantation set-up is feasible and will not introduce complications that can affect animal welfare. All trials proceeded successfully with no mice had displaying signs of excessive distress or injury from the implantation. Additionally, internal or core brain temperature of the implanted mice are well within the normal physiological levels of rodents, with the maximum limit at around 38°C (Yarmolenko et al., 2011; Mei et al., 2018). This is true whether the LED was activated or not. Along with the very minute increase in temperature during LED activation, temperature-related necrosis can be said to have not occurred. The relatively low internal body temperature can be attributed to the sleeping state of the recorded mouse (Sela et al., 2021). With the devices’ viability and novel features, many methodology changes can be explored. It is important to note that these statements are only applicable to short-term set-ups, those lasting for a few days per run. Nevertheless, the use of the device is still very safe for many methods.

The calcium signal imaging results have shown that there are perceivable qualitative differences in the fluorescence between the Formalin-injected and the PBS-injected group. The increase in signaling of the former group is attributable to nociception itself, as supported by behavioral data. Though the trend is not wholly consistent among mice, there a discernible pattern that can still be observed using our new device. In the images, fluorescent forms or shapes can be seen, especially in that of the DRN of the Formalin group. These forms are surely not individual neurons. They are much larger than the widest span (9–10 μm) of an average soma of neurons found in the selected sites. Light scattering through the tissue cannot be discounted as contributing to the size of the forms as seen on the images, but their effects are assumed to be minimal (Takehara et al., 2016). They are possibly neuronal clusters or ganglia and not glial cells because of the nature of GFP expression in GCaMP6 mice. The actual identity of these fluorescent forms is hard to ascertain because of the low resolution. This is an unavoidable limitation in the use of the CMOS imaging chip, especially the version we are currently using.

The formalin test is a method that can induce a bi-phasic response to pain: an early acute phase (0–5 min post injection) and a later tonic or inflammatory phase. The timing of the latter seems to differ among sources. The phase is mentioned to occur at around 15 min (Hunskaar et al., 1985; Hunskaar and Hole, 1987; Shibata et al., 1989; Rosland et al., 1990) until 60 min post injection (Manning and Mayer, 1995a). This is further complicated by the effect of environmental temperature on the potency of inflammation (Rosland, 1991; Tjølsen et al., 1992). The variability in the timing is reflected in the flattened second peak in the averaged behavior data of the Pain group (Figure 6, bottom).

The CeLC is a major component of the pain matrix. It has context-specific paradoxical roles of promoting hypoalgesia (Manning and Mayer, 1995a,b; Kang et al., 1998; Finn et al., 2003; Sabetkasaei et al., 2007; Yu et al., 2007; Veinante et al., 2013) and hyperalgesia. Hyperalgesia is accomplished during inflammation (Carrasquillo and Gereau, 2007; Veinante et al., 2013). Meanwhile, the DRN has also been shown to have an inflammation-specific antinociceptive function (Palazzo et al., 2004; Cucchiaro et al., 2005). Their inflammation-induced activation and the somewhat variable nature of the tonic phase timing might explain the persistent activity spikes of both sites in the Formalin group. This is reflective of the flattened, prolonged second peak, representing the tonic phase, in the Formalin group’s behavior data. This does not apply to the first distinct peak of the acute phase. Even if both the fluorescence and the behavior data are indicators of pain perception, the two data sets may not be total complements to each other because of the complex effects of formalin-induced nociception. Even so, together they still provide a holistic portrait for visualizing pain.

The qualitative visual data also reflects the results of the behavior analysis. The images of the DRN of the Formalin group display ROIs of higher fluorescence intensity compared to the PBS group. The difference is not fully apparent, especially for the CeLC in the latter half of the observation period. It was expected to fluoresce more prominently, following its central role in modulating pain perception. This weak response to pain stimulation can be attributed to the lateralization of the CeLCs of the two hemispheres. The right CeLC has been observed to be more responsive to nociception to a greater degree (Ji and Neugebauer, 2009; Allen et al., 2021), though this does not mean that the left CeLC is fully inactive (Allen et al., 2021). The descending architecture of the CeLCs are ipsilateral, especially the left one (Manning, 1998; Ji and Neugebauer, 2009). Since the study investigated the left brain hemisphere and induced pain contralaterally, the resulting calcium signaling could not have been strong. The fact that the devices have still detected pain-based trends in fluorescence between the two groups demonstrates its sensitivity.

Cross-correlation analysis showed that the Formalin-injected mice had more max cross-correlations at time lag 0. Though some ROIs displayed best lags at 1 or 2 frames, most other ROIs showed more synchronous activity. In contrast, the PBS-injected mice had more varying frame lags with several values higher than 1 frame delay/advance. The analysis indicated that there may be more asynchronous firing between CeLC and DRN neuronal firing in PBS-injected mice unlike the Formalin-injected mice. This suggests a coupling mechanism between or within the CeLC and the DRN during pain processing. The same mechanism is present in the adjacent cells of the dorsal root ganglion (DRG), a downstream neural pathway of the CeLC and the DRN, during inflammation and nerve damage (Kim et al., 2016). This synchronization was less apparent in naïve mice not exposed to pain.

Additionally, the highly variable time lag values of the cross-correlation coefficients of the ROIs portray a heterogenous population of neurons of differing roles in pain perception. For instance, the central amygdala can increase or decrease pain-related behavior depending on the cell type. Cells expressing protein kinase C-delta played a role in sensitization to nerve injury and increased pain response, while cells expressing somatostatin were inhibited and drove anti-pain behavior (Wilson et al., 2019). Furthermore, adjacent DRN 5-HT neurons are closely coupled and synchronized; however, non-adjacent 5-HT neurons are not. Also, there is a difference in the auto-correlograms of serotenergic and non-serotnergic neurons in the DRN, where non-5-HT cells are more irregular while 5-HT cells are more periodic (Wang and Aghajanian, 1982). Our analysis demonstrates such heterogenous interactions and behavior as well because ROIs within the same brain region, particularly the DRN of PBS mice, showed varying cross-correlograms. In addition, cross-correlation between CeLC and DRN ROIs had different time lags even if the mice were in the same treatment group.

The large gap in the tallied pain-based licking behavior of the groups confirms the successful execution of the formalin test, with the PBS group displaying none. This is reflected in the difference in calcium signaling between the Formalin and the PBS groups, though the peaks of the behavior tally graph and the fluorescence graph do not match after the initial phase. So, even though PBS Mouse 2 demonstrated relatively high CeLC brain activity, it is not indicative of any pain processing, based on behavior. This is further supported by and elucidated in the MI analysis between behavior and calcium signaling result.

MI is the amount of shared information between the two data, and shows how related they are with each other. A value of 0 indicates that the two data are independent. MI has several advantages such as being unbiased to the sample size, being model independent, being unrestricted to the data type, being able to detect linear and non-linear interactions, and being multivariate (Ross, 2014; Timme and Lapish, 2018). The analysis showed that the MI between calcium imaging of PBS mice and their licking behavior is close to 0 (Figure 10A). This means that the imaging data was not related to any mouse licking behavior in the PBS-injected mice. In contrast, the Formalin mice displayed significantly higher MI levels (Figure 10B).

Previous *in vivo* investigations on the mechanisms of pain, using calcium imaging, have mostly been done on the spinal cord level, as a necessity for less injurious methods of visualizing neural activity (Anderson et al., 2018; Miller et al., 2018; Xu and Dong, 2019). Our study is one of the first attempts to simultaneously image pain processing at two relevant brain regions *in vivo*, addressing the need for multi-site visualization for neuron-network studies (de Melo Reis et al., 2020). Because the protocols we used, from the lens-less CMOS-based fluorescence imaging device usage to the general experimental design modifications, are quite novel, there are many points for improvement that need to be addressed. Long-term double-implantation use of the devices can be explored. The methodology used to demonstrate the device, the formalin test, is very short in duration. It did not allow for the exploration of this aspect of device use. Determining the long-term viability of an implanted device would expand applications to studies involving chronic pain or multi-stage pain-conditioning experiments involving the same experimental animals. Retention of dual-implantation for an extended period can also more ensure better recuperation of the animals and give insight to potential changes to the integrity of the implanted devices. This can be done in future studies because of the parylene coating, which is usually used on medical implants. Because of the modular design of the device, components can easily be upgraded almost independent of each other. There are commercially available CMOS imaging chips of superior performance that can supplant what we have used. As of now, image quality derived from the CMOS chip we have used is relatively of inferior quality compared to that from lens systems. Because of the optics involved in a CMOS-based system vs. a lens system, images from the former will never match the resolving power provided by the latter. Even with an increase in pixel amount, the resolution will not necessarily improve. What this study had provided is a starting point for possible use of better components to better approach data quality of already established tools, but also providing unique advantages. Materials for the FPC substrate can also be made thinner, but more rigid, to ensure better implantation accuracy and safety. The blue-light filter used on our CMOS chips has been developed in our lab (Sunaga et al., 2014) and is still being improved upon through integration of additional filtering layers and better fabrication methods. Thus, there is proof of concept that the dual use of the device can work, but the device has potential still for refinement.

## Conclusion

We have shown that our developed implantable device can simultaneously be used to image two brain sites in mice while observing behavior, without hindrance or complications. The double implantation of the device was implemented across multiple experimental animals to successfully complete the modified formalin test. The collected fluorescence imaging data has been useful to some degree, enough to show trends and support established findings about pain processing. Though, there is a need for improvement, since at this point, the device is not sensitive enough to provide conclusive visuals. Proper selection of more active brain regions to image may better demonstrate its features. The properties of this implantable CMOS device will make it easy to apply on novel sites and configurations in future experiments.

## Data Availability Statement

The raw data supporting the conclusions of this article will be made available by the authors, without undue reservation.

## Ethics Statement

The animal study was reviewed and approved by the Nara Institute of Science and Technology (NAIST) Animal Committees.

## Author Contributions

All authors listed have made a substantial, direct and intellectual contribution to the work, and approved it for publication.

## Conflict of Interest

The authors declare that the research was conducted in the absence of any commercial or financial relationships that could be construed as a potential conflict of interest. The handling editor declared a past co-authorship with the authors.
